# Fluid Resuscitation for Refractory Hypotension

**DOI:** 10.3389/fvets.2021.621696

**Published:** 2021-03-10

**Authors:** Alexander Valverde

**Affiliations:** Department of Clinical Studies, Ontario Veterinary College, University of Guelph, Guelph, ON, Canada

**Keywords:** anesthesia, cardiac output, hypotension, sepsis, vasodilation

## Abstract

Hypotension is a common occurrence, especially in anesthetized patients and in critical patients suffering from hypovolemia due to shock and sepsis. Hypotension can also occur in normovolemic animals, anesthetized or conscious, under conditions of vasodilation or decreased cardiac function. The main consequence of hypotension is decreased organ perfusion and tissue injury/dysfunction. In the human literature there is no consensus on what is the threshold value for hypotension, and ranges from < 80 to < 100 mmHg for systolic blood pressure and from < 50 to < 70 mmHg for mean arterial blood pressure have been referenced for intraoperative hypotension. In veterinary medicine, similar values are referenced, despite marked differences in normal arterial blood pressure between species and with respect to humans. Therapeutic intervention involves fluid therapy to normalize volemia and use of sympathomimetics to enhance cardiac function and regulate peripheral vascular resistance. Despite these therapeutic measures, there is a subset of patients that are seemingly refractory and exhibit persistent hypotension. This review covers the physiological aspects that govern arterial blood pressure control and blood flow to tissues/organs, the pathophysiological mechanisms involved in hypotension and refractory hypotension, and therapeutic considerations and expectations that include proper interpretation of cardiovascular parameters, fluid recommendations and therapy rates, use of sympathomimetics and vasopressors, and newer approaches derived from the human literature.

## Introduction

### What Is Hypotension?

In simple terms, hypotension is a lower than normal arterial blood pressure. However, this definition does not imply when and at what specific values is hypotension harmful to the patient. Hypotension is a common occurrence, especially in anesthetized animals and in critical patients suffering from hypovolemia due to shock and sepsis.

Mean arterial blood pressure values of 60 mmHg or less and systolic arterial blood pressure values of 80 mmHg or less have been reported as indicative of excessive hypotension in animals, and ideally these pressures should be maintain above 80 mmHg and 100 mmHg for mean and systolic arterial blood pressure, respectively (1). However, arterial blood pressure values vary among species (Table 1), so the use of a common value as the threshold value for all species is not accurate and is best to keep in mind specific species values during the assessment of low arterial blood pressures. In addition, arterial blood pressure is consistently lower in the anesthetized than in conscious patients.

**Table 1:** Normal arterial blood pressure (mmHg) and cardiac index (mL/kg/min) in different species.

	**Conscious**				**References**
	**Systolic**	**Diastolic**	**Mean**	**Cardiac index**	
Human	120	80	93	71–86	(2)
Dog	150	98	115	165	(3, 4)
Cat	108–120	79–87	92–103	200	(5, 6)
	130–151	94–115	113–140	NR	(7)
	NR	NR	142–165	NR	(8)
Horse	147	92	115	60	(9)
	163	100	125	79	(10)
Cattle	155	105	123	119	(11)
Sheep	125	96	109	144	(11)
Goat	105	70	86	132	(11)
Rabbit	100	65	75	104–237	(12–15)

*NR, not reported*.

In humans, where studies have been completed with sufficient statistical power, there is still no consensus on what is the threshold value for hypotension to provide a widely accepted definition (16–18) and this is further complicated when adults are compared to pediatric patients (19) because arterial blood pressure is consistently lower in the latter population.

## Arterial Blood Pressure

### Physiology for Arterial Blood Pressure Control

The major function of the nervous system on the circulatory system is to control arterial blood pressure by allowing adequate and independent blood flow to the individual needs of tissues and organs, and regardless of the amount of blood that flows to any given tissue, arterial blood pressure remains constant (20).

**Figure 1:**
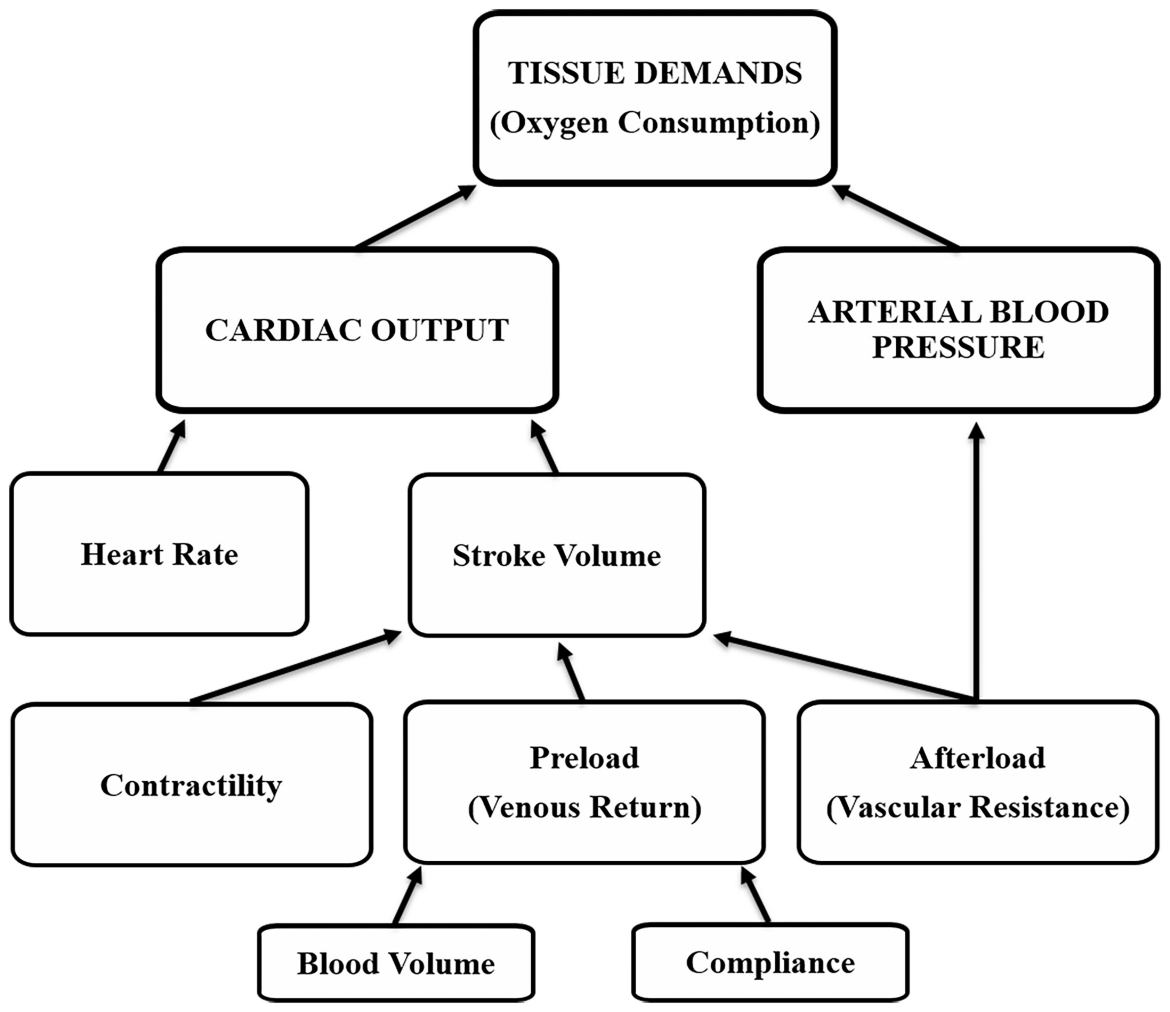
Tissue oxygen demands are met by cardiovascular factors that affect cardiac output and arterial blood pressure.

Arterial blood pressure control is necessary for short- and long-term situations, and it differs between. Short-term refers to acute changes (usually minutes to hours), whereas long-term refers to chronic changes (hours to weeks). Short-term control occurs through changes that affect both cardiac output and arterial blood pressure, and include variations in vascular resistance and compliance, contractility, heart rate and blood volume (Figure 1) (20). An example of short-term control are changes that occur during exercise in all or most of the factors (resistance, compliance, contractility, heart rate), to increase arterial blood pressure and cardiac output to meet tissue demands, but the increase in cardiac output exceeds that of arterial blood pressure because local muscle activity results in a decrease in vascular resistance (vasodilation) to improve blood flow while veins and small arterioles of other tissues exhibit a decrease in vascular compliance (constriction) to improve venous return (20). If arterial blood pressure and cardiac output do not meet tissue demands, there is less provision of nutrients and oxygen and the onset of anaerobic activity (21).

Individually, each of these factors result in specific changes in arterial blood pressure and/or cardiac output; however, with an intact nervous system it is the interaction between factors that determines the final cardiovascular effect. In simple terms, Ohm's Law [electrical current (I) through a conductor is proportional to voltage (V) and inversely proportional to resistance (R), (I = V ÷ R)] can be used to define the interaction between arterial blood pressure and cardiac output, where I is equivalent to cardiac output (CO), V is arterial blood pressure (ABP), and R is vascular resistance (VR), so that CO = ABP ÷ VR. For example, vascular resistance on its own can affect arterial blood pressure and cardiac output in opposite ways, a decrease in vascular resistance lowers arterial blood pressure and could increase cardiac output, whereas an increase in vascular resistance results in higher arterial blood pressure and limits cardiac output (20).

Volume administration increases cardiac output by increasing preload, and can potentially increase arterial blood pressure if the nervous system does not exert a reflex control over vascular resistance. This effect of volume administration without a change in arterial blood pressure is notable in conditions of normovolemia, where an increase in circulating volume results in a decrease in vascular resistance (20, 22, 23) and the compliance of the vascular system and tissues can store a significant volume, blunting any change in circulating volume (20, 22–25). If the decrease in vascular resistance was prevented during fluid administration in a normovolemic patient with a vasoconstrictor, then arterial blood pressure could also increase in conjunction with cardiac output (20). This is often observed with use of alpha-adrenergic drugs (vasoconstriction) but only if contractility can overcome the increase in vascular resistance (afterload) (26–29). The benefits of volume administration to increase arterial blood pressure are obvious in conditions where there is an ongoing deficit in circulating volume (hypovolemia).

An increase in heart rate can result in an increase in cardiac output and arterial blood pressure, but only if the circulating volume is ideal. A low circulating volume (hypovolemia) results in low venous return (preload) and a low right atrial pressure, even below 0 mmHg. The veins entering the chest will collapse with subatmospheric pressures and without proper preload the cardiac output and arterial blood pressure do not benefit from an increase in heart rate (20). Conversely, if preload is increased, an increase in heart rate is beneficial to cardiac output and arterial blood pressure, because it helps eject effectively the returning blood back into the circulation. However, an excessive increase in heart rate is not beneficial because diastolic time is shortened and decreases stroke volume, in addition to increasing the work of the heart, which weakens the heart contractility, all of which affect cardiac output negatively.

Long-term control of cardiac output and arterial blood pressure is the result of adaptation of baroreceptors within hours to days to changes in circulating volume and arterial blood pressure, of the instauration of higher vascular resistance that results in higher arterial blood pressure and minimal changes in cardiac output, and of an active participation of the kidney that responds to the increase in arterial blood pressure with endocrine and electrolyte mechanisms to control pressure and volume by increasing urinary output (20).

### Hypotension and Tissue Perfusion

Hypotension has been associated with decreased organ perfusion and injury/dysfunction, which in humans may include myocardial infarction (17, 30, 31), acute kidney injury (17, 31–34) and increased mortality (30, 35).

In general, most studies in humans have associated mean arterial blood pressures of ≤ 55 mmHg with adverse cardiac- and renal-related outcomes, and the duration of the hypotensive episode increases the risk of that adverse outcome (17, 18, 31, 34). Even at mean arterial blood pressures just < 80 mmHg, a hypotensive episode of more than 10 min can increase the risk of organ injury, and at lower pressures, less time is required for increased risk (18). Interestingly, improving cardiac index by 21% and mean arterial blood pressure from 65 to 85 mmHg with norepinephrine in septic human patients did not result in better renal function than in patients kept at the lower arterial blood pressure and cardiac index (36); likewise, in research dogs, acepromazine intramuscular administration as premedication resulted in a mean arterial blood pressure of 66 mmHg due to its vasodilatory effects, during isoflurane anesthesia for 2.25 h, lower than pressures in the same dogs after saline administration (87 mmHg), but creatinine levels, ALP-creatinine ratio in urine, and renal blood flow and glomerular filtration rate (GFR) assessed with scintigraphy were similar and within normal ranges in both groups (37).

Several aspects need to be considered to interpret hypotension. First of all, the threshold value for hypotension should not be generalized between conscious and anesthetized patients, since as stated before, values differed in the two situations and values also differ between species. A patient positioned in recumbency (dorsal, ventral, lateral) position, as it happens during anesthesia, offers the heart ideal work conditions because the effect of gravity is reduced by leveling all organs and tissues with the heart, and therefore changes to increase vascular resistance become less necessary in stable patients. In the standing position, gravitational forces result in lower pressures in the upper part of the body and higher pressures in the lower part of the body, which is counteracted by an increase in arterial vascular resistance and pulse rate, so that mean arterial blood pressure remains constant or slightly elevated throughout the body (38). In addition, in the standing patient, cardiac output decreases due to the increase in vascular resistance, and tissues can have a reduction in blood flow, including the kidney, which decreases GFR and increases arginine vasopressin (antidiuretic hormone) (38). General inhalant anesthesia and recumbency usually results in vasodilation and a decrease in vascular resistance, which helps maintain cardiac output if contractility is adequate, despite the reduction in mean arterial blood pressure. This could help explain why despite low mean arterial blood pressure in anesthetized patients, renal blood flow and function can remain normal in some studies (36, 37). Furthermore, the additional decrease in vascular resistance caused by drugs like acepromazine due to its alpha-blockade, allows for GFR and renal blood flow to be maintained within normal limits during general anesthesia, unlike conscious dogs maintained in lateral recumbency, where mean arterial blood pressures of < 80 mmHg decreased GFR but not renal blood flow, which was also impaired if mean arterial blood pressure was < 66 mmHg (39). These studies demonstrate that if the decrease in mean arterial blood pressure is the result of a decrease in vascular resistance, while contractility (cardiac output) is acceptable, an anesthetized patient can tolerate these pressures if not excessively low or for too long, and maintain adequate blood flow to tissues for their normal function.

It is also difficult to conclude when hypotension is significant enough to result in an adverse outcome, which is further confounded by how hypotension is defined in any study. Findings from different studies cannot be generalized because small changes in the selected threshold values for hypotension among human studies can result in wide differences in the incidence of intraoperative hypotension (5–99%) and do not necessarily reflect organ dysfunction as a consequence of hypotension (16). More importantly, the variation in adverse outcomes as it relates to hypotension can be explained in part by the health status of the patient [American Society of Anesthesiologists (ASA) classification] and whether pre-existing hypovolemia or cardiac disease are the primary causes, since morbidities and mortality are associated with older age and higher ASA status risk in human patients (17, 18, 30, 31, 33, 35).

Studies in healthy dogs and foals under research conditions, where hypotension was induced by deep anesthesia with inhalant anesthetics in normovolemic conditions, resulted in no adverse outcomes despite mean arterial blood pressures of 24 mmHg for at least 15 min in foals (26) and 46 mmHg for at least 45 min in dogs (23), although pressures were normalized in those animals before recovery from anesthesia.

## Refractory Hypotension

Hypotension that persists, despite appropriate therapeutic measures that consistently correct it, probably exhibit refractory hypotension. Refractory hypotension is most commonly encountered under conditions of shock due to hypovolemia or sepsis, but can also include cardiogenic, neurogenic, and anaphylactic shock (40). Refractory hypotension and shock develops in nearly 6% of critically ill human patients (41) and mortality rates are usually > 40–50% because of progressive hypotension (42, 43).

### Hypovolemic Shock

Hypovolemic (hemorrhagic) shock is due to a reduced intravascular volume (preload), which results in low cardiac output and arterial blood pressure. In human medicine hypovolemic shock is rated into four classes, from least to most critical, according to the “Advanced Trauma Life Support (ATLS®)” guidelines of the American College of Surgeons. The patient is allocated to one of the classes according to degree of blood loss (< 15% in Class I, 15–30% in Class II, 30–40% in Class III, and > 40% in Class IV) and degree of alteration of vital signs, including pulse rate, blood pressure, pulse pressure, respiratory rate, mental status, and urinary output (44). However, not every trauma patient fits the description of any single class and only in classes III and IV is hypotension a clinical sign (44).

In conscious, splenectomized research dogs, removal of 43 mL/kg of blood [53% of blood volume; (45)] in 10 min resulted in an immediate drop in mean arterial blood pressure from 106 mmHg to 25 mmHg and a gradual recovery over 72 min to a decompensated mean arterial blood pressure of 61 mmHg (46). During this period, dogs were untreated and heart rate increased from 81 beats/min before hemorrhage to over 186 beats/min, and both plasma total protein and packed cell volume decreased. Over the next hour, mean arterial blood pressure decreased again to the initial drop and heart rate was over 220 beats/min, to which the dogs succumbed to death (46).

The response to blood loss is different under conditions of general anesthesia, both with inhalant and injectable anesthetics, because these drugs inhibit, in a dose-dependent fashion, baroreceptor reflex and sympathetic activity, which interferes with normal short-term control (47–49). In addition, blood loss in itself results in decreased requirements of inhalant anesthetics (50), which deems the patient at a deeper level of anesthesia during the hypovolemic episode and further inhibits any sympathetic response. In anesthetized research dogs, maintained at an end-tidal isoflurane concentration of 1.27 to 1.74% [~1.0–1.3 times the minimum alveolar concentration (MAC)] to induce a mean arterial blood pressure to 65 mmHg, removal of ~25 mL/kg of blood (31% of blood volume) over 30 min resulted in inhalant anesthetic dose-dependent effects: no change in arterial pressure with lighter anesthesia to a drop to 45 mmHg with deeper anesthesia, heart rate can remain unchanged or increase slightly with lighter anesthesia and decrease slightly with deeper anesthesia, cardiac index and stroke volume may not change with lighter anesthesia and decrease with deeper anesthesia, systemic vascular resistance not change or increase slightly, and total protein and packed cell volume had not changed with the bleeding (51, 52). A similar blood loss of 30 mL/kg in one study decreased MAC by 16% (50) and it is likely that some of the observed changes are also influenced by the increased MAC multiple (if MAC decreases by 16% and the end-tidal inhalant concentration is not adjusted, MAC becomes 1.16% higher); therefore, under clinical conditions is important to consider adjusting the anesthetic plane during hemorrhage to avoid the contribution of a higher MAC multiple on blood loss and its negative effect on the inhibition of baroreflexes and sympathetic activity (53).

### Septic Shock

Septic shock is discussed in depth in the chapter “Fluid Therapy in Dogs and Cats with Sepsis.” In brief, septic shock is a type of distributive shock, in which infection results in organ dysfunction due to a dysregulated response from the patient that results in injury to its own tissues and organs (43). Patients with sepsis exhibit hypotension due to peripheral vasodilation, so septic shock is also known as vasodilatory shock or a state of vasoplegia, and is the most common form of shock encountered in intensive care units (43, 54). The pathophysiological mechanisms, responsible for vasoplegia and decrease vascular responsiveness in septic shock, result in refractory hypotension and are also common to hypovolemic shock, cardiogenic shock and anaphylactic shock (40).

According to the latest consensus for sepsis and septic shock in humans (Sepsis 3.0), patients clinically exhibit persistent hypotension that requires of vasopressors to maintain a mean arterial blood pressure ≥ 65 mmHg and a serum lactate level > 2 mmol/L, despite adequate volume resuscitation and the absence of hypovolemia (43). In addition, despite adequate tissue perfusion achieved with ideal mean arterial blood pressure and normovolemia, cells undergo conditions of dysoxia (i.e., inability to match oxygen demand with oxygen supply), which results in organ dysfunction and high lactate levels and results in mortality rates in excess of 40% in human patients (43) and veterinary patients (55–64).

### Mechanisms Responsible for Refractory Hypotension

The vasodilation that results from sepsis is the result of several factors that enhance vasodilatory- and inhibit vasoconstrictor mechanisms. These include an unregulated synthesis of nitric oxide, a potent vasodilator, in response to cytokines released during septic and hemorrhagic shock. In dogs with septic peritonitis, levels of the inflammatory cytokines C-C motif chemokine ligand 2 (CCL2) and interleukin 6 (IL-6) were significantly higher than in non-septic dogs (64), which contributes to migration and infiltration of macrophages into the area of insult (65). Nitric oxide is produced from L-arginine by nitric oxide synthase, present in endothelial cells, and diffuses into the underlying smooth muscle to activate soluble guanylate cyclase and cGMP production that dephosphorylates myosin, via the myosin light chain phosphatase, causing vasorelaxation (54, 66, 67).

Another mechanism is the result of conditions of local acidosis, with hydrogen ions and lactic acid production from anaerobic metabolism (43, 56, 60, 64, 68, 69). These conditions result in opening of ATP-sensitive potassium channels present in the plasma membrane of vascular smooth muscle and allow an efflux of potassium that hyperpolarizes the plasma membrane and prevents calcium from entering the cell; without intracellular calcium, the phosphorylation of myosin is prevented and the smooth muscle does not contract (54, 66, 70). Metabolic acidosis also promotes overproduction of nitric oxide (70).

The prostaglandin prostacyclin is found in increased concentrations in conditions of septic shock (71). Prostacyclin forms from arachidonic acid from the vascular endothelium by the actions of the cyclooxygenase enzymes and prostacyclin synthase, and once formed it binds to the prostacyclin receptor (IP) and acts on a G-protein coupled receptor which activates adenyl cyclase and stimulates the formation of c-AMP from ATP, which activates protein kinase A and prevents increases in cytoplasmic Ca^2+^, preventing vasoconstriction and resulting in vasodilation (67).

A final mechanism involves depletion of vasopressin reserves from the neurohypophysis (66). Vasopressin is a potent vasoconstrictor by acting on vasopressin-type 1a receptors (V_1a_) present in vascular smooth muscle and helps with short-term control of arterial blood pressure under conditions of hemorrhage or sepsis, but this process requires of high circulating concentrations of vasopressin that depletes the stores within 1-h under conditions of profound baroreflex stimulation (54, 66). Interestingly, vasopressin concentrations required for its antidiuretic effect are much lower and still effective under conditions of shock (66).

## Treatment

**Figure 2:**
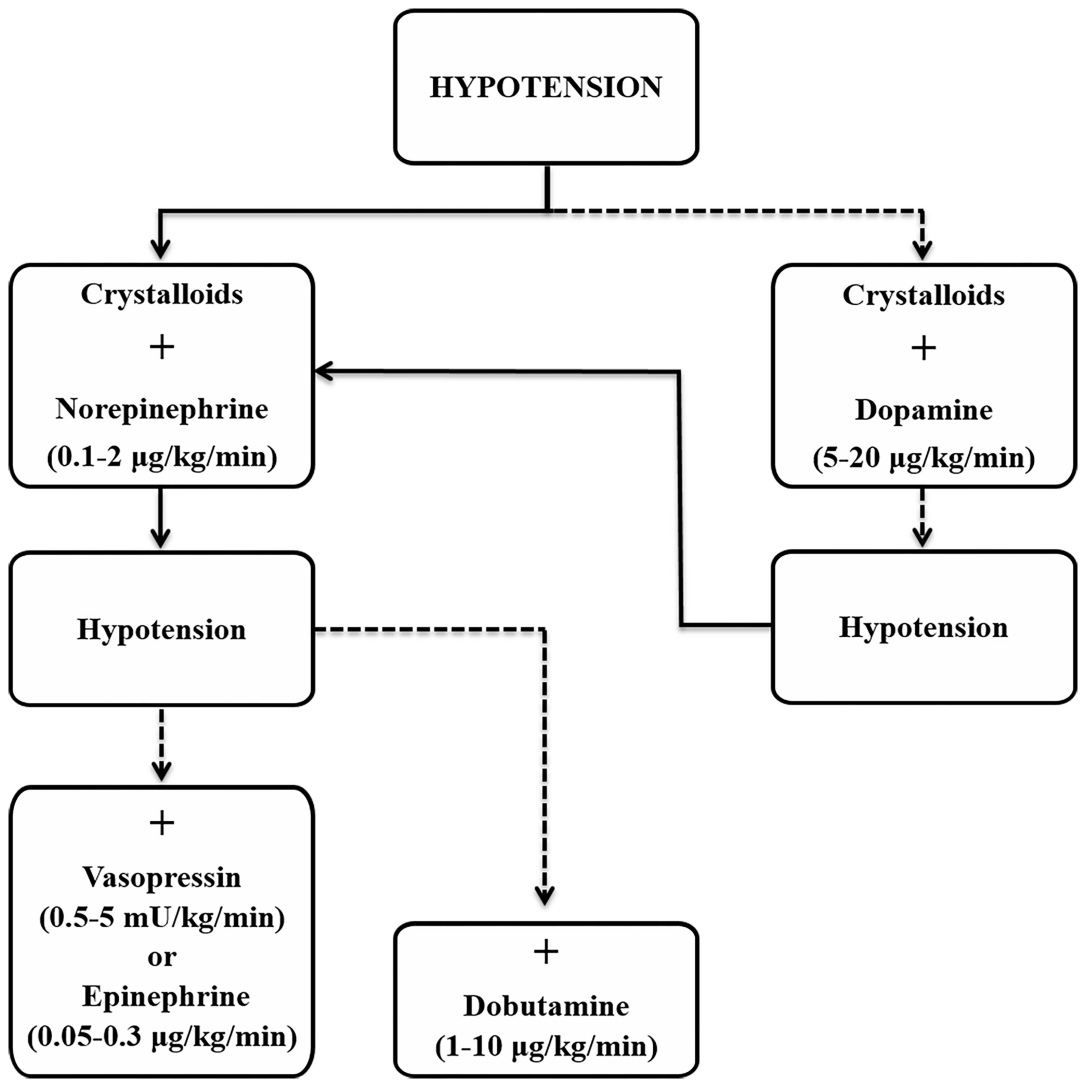
Suggested approach for the treatment of hypotension based on current guidelines for management of sepsis and septic shock (72). Solid line, strong and preferred recommendation; dashed line, weaker recommendation.

The most important step to treat refractory hypotension is to determine the primary cause(s). These include two main reasons, hypovolemia and/or sepsis, and within them the whole spectrum of possibilities, from blood loss, dehydration, sympathetic inactivity or inability to respond, metabolic acidosis, and endotoxemia. Sepsis however, is the most common cause of refractory hypotension due to the vasodilatory effects that result from it (42, 43, 54). The aim of treatment is to overcome the vasodilation that results in hypotension, which is common to classes III (> 30–40% blood loss) and IV (> 40%) of shock (44). An algorithm is provided (Figure 2) to help in the decision process during treatment, with an emphasis on short-term control by manipulation of the factors that influence cardiac output and arterial blood pressure, including vascular resistance and compliance, contractility, heart rate and blood volume.

Monitoring of cardiovascular function to assess the condition of the patient and response to treatment is primordial. Arterial blood pressure is readily monitored by non-invasive methods; unfortunately they are inaccurate and unreliable in hypotensive patients (73). Direct arterial blood pressure monitoring is often pursued in critical patients, especially if under general anesthesia, and provides accurate values that can be continuously monitored to assess the effectiveness of fluid therapy when used with dynamic cardiovascular variables such as pulse pressure variation, stroke volume variation and systolic pressure variation, to better define fluid- responsive from non-responsive patients (see chapter “What are the most effective methods for monitoring IV fluid therapy?”).

### Vasopressors

This review starts with vasopressors as the first line of therapy since the basic pathology is the decrease in vascular resistance. However, a combination of at least vasopressors and fluids should be instituted simultaneously for better success in improving arterial blood pressure. The most recent guidelines for management of sepsis and septic shock in people, from 2016, recommends an initial target mean arterial pressure of 65 mmHg in patients with septic shock requiring vasopressors (72).

Vasopressor therapy aims at improving tissue perfusion through vasoconstriction and improved contractility. Norepinephrine, dopamine, epinephrine and phenylephrine are catecholamines used for their vasoconstrictor actions through the alpha-1 receptor, and inotropic actions through the beta-1 receptor, except for phenylephrine that lacks the latter. Arginine vasopressin (vasopressin) is also used for its vasoconstrictor effects, through its V_1a_ receptor action.

Vasoconstrictor actions of alpha-adrenergic drugs on the alpha-1 receptor and of vasopressin on the V_1a_ receptor are linked to G-protein-coupled receptors that result in smooth muscle contraction. Attachment of these agonists to the respective receptors triggers the stimulation of phospholipase C, which generates the production of inositol triphosphate (IP_3_) and diacylglycerol (DAG). These two substances interact between them to promote smooth muscle contraction; IP_3_ diffuses into the cell to act on an IP_3_-sensitive Ca^2+^ channel on the surface of the endoplasmic reticulum, facilitating the release of Ca^2+^ into the cytoplasm, which stimulates the calmodulin (CM) pathway by forming a Ca^2+^-CM complex that activates the myosin light-chain kinase and with the presence of ATP, it phosphorylates the myosin light chain and causes vasoconstriction (54). DAG remains in the cell membrane and stimulates the influx of Ca^2+^ into the cytoplasm through a Ca^2+^ channel; this additional Ca^2+^ with the Ca^2+^ released by the actions of IP_3_ also stimulates the CM pathway. In addition, the Ca^2+^ from DAG's actions stimulates the janus kinase 2 (JAK2) pathway, responsible for activating rho kinase, which prevents smooth muscle relaxation from occurring by inhibiting the myosin light chain phosphatase, as well as helps release reactive oxygen species, which are useful in increasing the sensitivity to Ca^2+^ and leads to additional stimulation of rho kinase (54).

Inotropic actions of beta-adrenergic drugs on the beta-1 receptor are also linked to G-protein-coupled receptors that trigger the stimulation of adenylyl cyclase to generate the production of cAMP and activates protein kinase A (PKA), which activates L-type Ca^2+^ channels, phospholamban, sarcoplasmic reticulum ryanodine receptor channels (SR), cardiac troponin I (cTnI) and myosin binding protein (MyBP-C) (74, 75). Phospholamban phosphorylation removes its inhibitory effect on SR Ca^2+^ uptake and increases L-type Ca^2+^ current and SR Ca^2+^ cycling to allow for positive inotropic effects, with the contribution of PKA-dependent cTnI phosphorylation (74).

Patients with refractory hypotension under general anesthesia should be supported for contractility through beta-adrenergic inotropic effects because of the vasodilation from inhalant anesthetics and their negative dose-dependent effects on contractility (47, 53). Ideally, a drug with alpha-1 and beta-1 effects should be used to improve both arterial blood pressure and cardiac output, but not at the expense of excessive vasoconstriction that may negatively affect cardiac output. For example, increasing mean arterial pressure to 85 mmHg in patients with septic shock did not improve renal values and metabolic markers (lactate, oxygen consumption) from those already achieved at 65 mmHg (36).

The guidelines for management of sepsis and septic shock have published recommendations on the different types of vasopressors that should be used for resuscitation upon reviewing the literature; the higher the quality of evidence, the more likely a strong recommendation is issued (72). According to the guidelines, norepinephrine is strongly recommended/moderate quality of evidence as the first line-drug (72). Norepinephrine, through its alpha- and beta-1 adrenergic actions, can increase blood pressure and cardiac output (26).

Vasopressin has been considered the second line-drug due to its catecholamine-sparing properties (40, 54). The vasoconstriction and increase in vascular resistance caused by vasopressin can decrease cardiac output (26), and the recent guidelines recommend adding either vasopressin (weak recommendation/moderate quality of evidence) or epinephrine (weak recommendation/low quality of evidence) to norepinephrine to reach the target mean arterial pressure, or adding vasopressin (weak recommendation/moderate quality of evidence) to decrease the norepinephrine dose (72).

Vasopressin due to its actions on V_1a_ receptor does not result in arrhythmias and can increase systemic arterial blood pressure even in the presence of acidemia (76) and has been recommended in refractory shock due to a relative vasopressin deficiency and to benefit from a norepinephrine-sparing effect (40, 77). However, vasopressin can compromise splanchnic and peripheral tissue perfusion under conditions of hypovolemia and these negative effects should be considered (76).

Other catecholamines have been used according to availability, but both norepinephrine and the non-catecholamine vasopressin are considered superior (40, 72). Epinephrine delays normalization of arterial pH and lactate concentrations from exaggerated aerobic glycolysis through Na^+^K^+^ ATPase stimulation within the muscles, which may interfere with interpretation of a patient's proper responsiveness to treatment and is likely to indicate anaerobic metabolism (78, 79). The current guidelines for management of sepsis and septic shock do not state a specific recommendation for epinephrine on its own, but added to norepinephrine, despite no difference in mortality rate when compared to norepinephrine, although adverse drug-related events were more common with epinephrine (72). In the previous guidelines from 2012, a weak recommendation / moderate quality of evidence was issued for epinephrine (80).

Dopamine is less efficient at normalizing arterial blood pressure than norepinephrine and it has been associated with arrhythmias than norepinephrine (42), most likely because vasoconstrictor effects of dopamine are achieved with higher infusion rates, which also exacerbate beta-1 adrenergic effects (81). In addition, mortality is higher than in patients receiving norepinephrine (42). Dopamine is recommended by the guidelines for management of sepsis and septic shock as an alternative vasopressor to norepinephrine only in highly selected patients (e.g., low risk of tachyarrhythmias and absolute or relative bradycardia) with a weak recommendation / low quality of evidence (72). There is also a strong recommendation/high quality of evidence against the use of low-dose dopamine for renal protection (72).

Phenylephrine is a pure alpha-1 agonist; therefore only vasoconstriction is expected from it, which may be detrimental in hypovolemic patients that also exhibit a reduction in cardiac output, unless contractility is supported with beta-1 agonists (81). Septic patients treated with phenylephrine had a higher mortality rate than patients treated with norepinephrine (82) and current recommendations from the guidelines for management of sepsis and septic shock state that phenylephrine use should be limited until more research is available (72).

Higher doses of catecholamines and/or vasopressin are usually required in refractory hypotension from a decreased response to endogenous or exogenous vasoactive substances such as angiotensin II and vasopressors (83). Tachyphylaxis occurs with high doses and repetitive exposure to alpha-adrenergic drugs, because the alpha-1 receptors are desensitized and internalized (84).

The use of higher doses results in a higher incidence of adverse effects, including arrhythmias (42, 79). In attempts to modulate individual effects and reduce adverse effects, norepinephrine has also been combined with dobutamine (78, 79). Dobutamine increases cardiac output through its beta-1 adrenergic effects (inotropy), which increases arterial blood pressure under ideal conditions of vascular tone since it lacks alpha-adrenergic effects (26). The guidelines for management of sepsis and septic shock suggest using dobutamine in patients who show evidence of persistent hypoperfusion despite adequate fluid loading and the use of vasopressor drugs, under a weak recommendation/low quality of evidence (72).

Terlipressin is a prodrug to its metabolite lysine vasopressin; both are more selective and have a longer action than vasopressin for the V_1_ receptor for the specific vasoconstrictor effects, but its use is still under investigation for refractory hypotension (40, 54). Selepressin is another V_1_ receptor agonist with a short duration of action, that has demonstrated positive effects in cardiovascular variables and fluid balance to prevent lung edema in an ovine model of septic shock (85) and is currently undergoing investigation.

Standard doses of sympathomimetics and vasopressin used to treat hypotension include 1–10 μg/kg/min for dopamine, 1–10 μg/kg/min for dobutamine, 0.1–2 μg/kg/min for norepinephrine, 0.05–0.1 μg/kg/min for epinephrine, 0.5–2 μg/kg/min for phenylephrine, and 0.3–5 mU/kg/min for vasopressin (81). In human patients with refractory hypotension, higher doses than those mentioned above for each of these drugs are often necessary in attempts to manage their cardiovascular derangement (41, 42, 86); from a prognostic point of view, the need for higher doses of vasopressors is associated with unfavorable outcomes (41, 86).

### Fluid Therapy

Isotonic crystalloids are recommended as the initial fluid of choice for shock reversal in septic patients, since the time to reach preset values of mean arterial pressure, central venous oxygen saturation, normalization of lactate levels and discontinuation of vasopressors, was achieved as quickly as in patients resuscitated with a combination of synthetic colloids (hydroxyethyl starch or gelatin) and crystalloids (32, 72, 87, 88). One disadvantage of synthetic colloids is that despite their volume expansion/retention properties, often result in higher incidence of impaired renal function than crystalloids and required volumes of resuscitation are only marginally lower than for crystalloids (32). In addition, using starches, dextrans, albumin, fresh frozen plasma, or gelatins *vs*. crystalloids makes little or no difference in mortality of critically ill patients (87, 88). Isotonic crystalloids also resulted in similar outcomes when compared to hypertonic crystalloids (88).

The guidelines for management of sepsis and septic shock in people recommends fluids as follows: a strong recommendation/moderate quality of evidence for crystalloids; a weak recommendation/low quality evidence for use of either balanced crystalloids or saline; a weak recommendation/low quality of evidence for albumin in addition to crystalloids; a strong recommendation/high quality of evidence against hydroxyethyl starches; and a weak recommendation/low quality of evidence for crystalloids over gelatins (72).

Studies in normovolemic and normotensive dogs, maintained at useful clinical inhalant anesthetic concentrations to allow surgery (1.1–1.25 MAC), arterial blood pressure values and the effects on packed cell volume and total protein were similar in groups receiving no fluid therapy or up to 20 mL/kg/h of isotonic crystalloids (24, 25), because fluid balance dynamics and cardiovascular function are well-preserved in those conditions of light anesthetic planes (89, 90).

In hypotensive models using healthy research dogs that were normovolemic and maintained at a systolic arterial blood pressure of 80 mmHg with a deep level of isoflurane anesthesia [3.1–3.4% end-tidal (2.4–2.8 MAC)], isotonic crystalloids, at fast and high volume infusion rates used for shock of 80 mL/kg for up to 1-h did not improve blood pressure, whereas the colloid hetastarch administered at a rate of 80 mL/kg/h to maximum volume 40 mL/kg, restored systolic pressures to within 10% of baseline values predetermined at 1.6–1.7% (1.3 MAC) in 66% of cases (22). In a similar study, research dogs maintained also at 3% (2.3 MAC), arterial blood pressure did not improve with the administration of isotonic crystalloids also infused at a high volume rate of 60 mL/kg/h for 45 min, until the depth of anesthesia was decreased to 1.6% while the fluid rate continued for another 15 min (23).

Normovolemic patients that exhibit hypotension under general inhalant anesthesia do not have increases in arterial blood pressure with high volume fluid rates of isotonic crystalloids, and are also less responsive to colloids, because the infused volume results in a decrease in systemic vascular resistance (22, 23). Despite the hypotension, cardiac output, lactate, venous and arterial pH, and mixed venous oxygen saturation remained within acceptable levels and arterial blood pressure did not decrease any further in these research models (22, 23). According to Ohm's law (ABP = CO ^x^ VR), arterial blood pressure was maintained as a result of an improvement in cardiac output from the expansion in blood and plasma volume (22, 23). Interestingly, with crystalloid administration and under the conditions of deep anesthesia, heart rate and stroke volume did not change in both studies (22, 23). Since cardiac output is also the product of both (CO = HR ^x^ SV), an increase in myocardial contractility is likely responsible for the improvement in cardiac output, as a result of the expanded circulating volume that decreases viscosity from hemodilution of the packed cell volume and total protein, which in association with the lower vascular resistance facilitates the work of the heart, even if arterial blood pressure does not change (22, 23). In this regard, the guidelines for management of sepsis and septic shock only recommend (strong recommendation/high quality of evidence) red blood cell transfusion if the hemoglobin concentration decreases to < 7.0 g/dL in the absence of extenuating circumstances, such as myocardial ischemia, severe hypoxemia, or acute hemorrhage (72).

Findings from healthy research dogs should be extrapolated cautiously to patients that suffer from refractory hypotension and shock. The expectations and benefits of fluid therapy in normalizing arterial blood pressure differ in normovolemic *vs*. hypovolemic patients. The effects of anesthetic drugs and depth of anesthesia should also be considered. Hypovolemic, septic patients exhibit refractory hypotension and low cardiac output, resulting in long lasting hypotension and severely compromised tissue perfusion that may become irreversible.

The benefit of fluid therapy in reestablishing cardiovascular variables in hypovolemic septic patients is limited without vasopressors. The use of vasopressors should always accompany fast and high volume rates of isotonic crystalloids in these patients, and should include frequent monitoring for hemodilution and decreases in colloid oncotic pressure, because these patients are likely to have impaired oxygen content and delivery, altered fluid transmembrane dynamics and predisposition to edema, all of which affect tissue integrity (91). The most recent guidelines for management of sepsis and septic shock strongly recommends/low quality of evidence that at least 30 mL/kg of IV isotonic fluids be given within the first 3 h and that frequent reassessment of cardiovascular variables (heart rate, blood pressure, arterial oxygen saturation, respiratory rate, temperature, urine output, and others as available) (72).

### Other Drugs

#### Hydrocortisone

Cortisol released by the hypothalamic–pituitary–adrenal axis is important in mediating vasoconstriction through regulation of vascular smooth muscle sensitivity to the endogenous catecholamines norepinephrine and epinephrine and the hormone angiotensin II, and through regulation of cytokines. Mechanisms involved in restoring vascular tone include an increase in alpha-adrenergic receptor gene expression (92); inhibition of the arachidonic acid cascade (93, 94), preventing prostacyclin formation and its vasodilatory effects (67); genomic inhibition of the nuclear translocation of the transcription factor NF-κB, responsible for expression of pro-inflammatory genes for cytokines and chemokines (95); and inhibition of expression of inducible nitric oxide synthase from endothelial cells, halting nitric oxide production and inhibiting vasodilation (96, 97). Hydrocortisone provides both glucocorticoid and mineralocorticoid coverage.

The current guidelines for management of sepsis and septic shock suggests against the use of IV hydrocortisone to treat septic shock patients if adequate fluid resuscitation and vasopressor therapy are able to restore hemodynamic stability. If this is not achievable, IV hydrocortisone at a dose of 200 mg per day is suggested (weak recommendation/low quality of evidence) (72).

In patients receiving corticosteroid therapy for chronic autoimmune or inflammatory diseases and in patients receiving replacement therapy because of chronic primary or secondary adrenal insufficiency, it is important that they continue receiving their current dose of corticosteroids to maintain cardiovascular function, especially if general anesthesia is planned. If the patient exhibits refractory hypotension, supplemental hydrocortisone therapy should be considered.

#### Angiotensin II

Angiotensin II has been recommended due to its actions on the AT1 receptor, a G-protein-coupled receptor, and stimulates the same transduction pathways as vasopressors through activation of phospholipase C for the generation of IP_3_ and DAG and stimulation of the JAK2 pathway (21), all of which result in vasoconstriction, aldosterone secretion and vasopressin release (40).

Angiotensin II was evaluated in a double blind, randomized, controlled trial in patients with vasodilatory shock that were not responsive to high doses of vasopressors (98). In this study, patients receiving angiotensin II in combination with the vasopressor had lower requirements for vasopressors, including norepinephrine, and a higher mean arterial blood pressure than the placebo group receiving only the vasopressor; however, mortality rate and adverse effects were similar between the two groups (98). The dose used in this study for angiotensin II was 20 ng/kg/min for the first 3 h, and it was lowered in many instances due to an improvement in the mean arterial blood pressure (98).

Angiotensin II is not mentioned in the most current guidelines for management of sepsis and septic shock (72) because the study by Khanna et al. (98) is contemporary and was not available.

#### Methylene Blue

Methylene blue inhibits nitric oxide synthase, preventing the production of nitric oxide and cGMP, therefore vasodilation does not occur (99). *In vitro*, methylene blue also suppresses the production and release of prostacyclin, in both the presence and absence of pro-inflammatory cytokines, in dog renal artery smooth muscle cells, which may also contribute to prevent vasodilation by inhibiting the formation of c-AMP, which normally would prevent cytoplasmic Ca^2+^ from causing vasoconstriction (100).

Methylene blue has been used successfully at decreasing mortality in vasodilatory shock after cardiopulmonary bypass at IV doses of 1–2 mg/kg to counteract refractory hypotension that requires of high inotropic support (99). However, its use in vasodilatory shock due to sepsis is not yet established and is not mentioned in the current guidelines for management of sepsis and septic shock (72).

#### Alpha-2 Agonists

Sedation is often required in human septic patients. Dexmedetomidine has been used for this purpose and spiked interest due to its central and peripheral actions on sympathetic activity and vascular tone. Dexmedetomidine causes vasoconstriction and an increase in mean arterial pressure through its post-synaptic alpha-1 and−2 adrenergic actions, but it can be used also as an antihypertensive drug due to its presynaptic alpha-2 actions that induce a negative feedback on norepinephrine secretion, which causes hypotension (101). However, in rats induced to septic shock, dexmedetomidine administration increased the response to exogenous norepinephrine (102), indicating that it restores the responsiveness to alpha-1 agonists.

In human patients with septic shock and initially maintained sedated with propofol and remifentanil, while receiving norepinephrine to maintain adequate mean arterial blood pressures, had higher norepinephrine requirements than when dexmedetomidine was used to replace the propofol, and norepinephrine requirements increased again when sedation was switched back to propofol (103).

The use of dexmedetomidine is not mentioned in the current guidelines for management of sepsis and septic shock (72).

In summary, refractory hypotension should be addressed aggressively to reestablish arterial blood pressure control and blood flow to tissues/organs. Therapeutic actions include fluid therapy with the use of sympathomimetics and vasopressors that help overcome or at least minimize the ongoing state of vasoplegia, In anesthetized patients, the vasodilatory effects of inhalant anesthetics contribute to the hypotension and anesthesia time should be reduced if at all possible, as well as consider the use of balanced techniques that could help avoid excessive inhibition of sympathetic activity.

## Author Contributions

AV: manuscript writing.

## Conflict of Interest

The author declares that the research was conducted in the absence of any commercial or financial relationships that could be construed as a potential conflict of interest.
